# Influence Mechanism of Foamed Concrete Coating Thickness on the Blast Resistance of RC Walls

**DOI:** 10.3390/ma15165473

**Published:** 2022-08-09

**Authors:** Wei Shang, Zhengxiang Huang, Xudong Zu, Qiangqiang Xiao, Xin Jia

**Affiliations:** School of Mechanical Engineering, Nanjing University of Science and Technology, Nanjing 210094, China

**Keywords:** blast resistance, RC wall, foamed concrete, coating thickness, energy absorption

## Abstract

How to effectively reduce the damage of frequent accidental explosions and explosion attacks to existing walls is an important concern of the blast resistance field. In the present study, the influence of the foamed concrete (density 820 kg/m^3^, water-cement ratio 0.4) coating thickness on the blast resistance of a 120 mm RC (reinforced concrete) wall was studied through blast experiments, numerical simulations, and shock wave theory. Results show that the influences of foamed concrete on the blast resistance of RC walls are jointly decided by the stress drop caused by impedance effect and exponential attenuation and the stress rise caused by high-speed impact compression. The coating thickness mainly affects the foam concrete’s fragmentation degree and stress attenuation. A lower critical coating thickness exists in foamed concrete-coated RC walls. The blast resistance of the RC wall will decrease when the coating thickness is less than that value. The lower critical coating thickness is related to the intensity of blast load and the energy absorption capacity of foamed concrete, and it can be predicted by monitoring the explosive stress and energy incident to the RC wall.

## 1. Introduction

The frequent accidental explosions and explosive attacks in recent years often cause large-scale damage to buildings, accompanied by huge casualties and economic losses [1,2]. The RC (reinforced concrete) wall mainly bears lateral loads and is one of the most vulnerable parts of the building under blast load. Two major hazards may occur when the blast is loaded onto the RC wall: First, the spalling fragments generated at the back side of the wall will damage the equipment and personnel behind the wall; second, the bearing capacity of the wall decreases after local damage, thereby posing a risk of collapse [3]. Therefore, how to effectively weaken the blasting damage to existing RC walls and difficult research on the blast resistance design of buildings are a focus.

Foamed concrete is a lightweight porous building material, which can be constructed by cast-in-place or masonry, with the advantages of low price, simple maintenance, and suitability for large-scale application [4]. At present, foamed concrete has been widely used in road bridge engineering, backfill engineering, ocean engineering, military engineering, and many other fields [5]. Due to the foamed concrete being fragile, it can absorb a lot of energy under blast loading, which stimulates great research interest of scholars in the field of engineering protection [6]. In terms of blast resistance, Kolluru et al. [7] and Nian et al. [8] simulated the dynamic response of foamed concrete circular plate and cylindrical rod specimens under blast load through shock tube experiments. Results showed that foamed concrete mainly absorbed energy in the way of fragmentation, which can be used to weaken the blast wave transmitted to the main structure. Edward et al. [6] and Monir et al. [9] analyzed the relationship between the microscopic characteristics of foamed concrete and its fragility. Results indicated that the pores make foamed concrete fragile. Compared with traditional concrete, foamed concrete formed lighter and low-velocity fragments under blast loading, and the lethality of fragments was greatly reduced. Shang et al. [10] studied the influence of explosion distance and thickness on the fragmentation and energy absorption characteristics of foamed concrete slabs through blast experiments and fractal theory. Wang et al. [11] and Tian et al. [12] analyzed the dynamic response law of foamed concrete steel plate/shell structure under different explosion conditions based on blast experiments. Results showed that the main influencing factors of foamed concrete cushioning effect were the load intensity, foamed concrete’s thickness, density, and compressive strength. Zhao et al. [13] and Yu et al. [14] studied the influence of additional foamed concrete layers on the blast resistance of the tunnels through numerical simulation. The results showed that the tunnel deformation was significantly reduced under the cushioning effect of foamed concrete. It can be concluded from the above research that foamed concrete can be used as a cushioning layer to reduce the damage of blast load to the structure. Fragmentation is the main energy absorption form of foamed concrete.

Thickness is one of the important structural parameters of foamed concrete cushions, and the influence of thickness has always been a concern. Nian et al. [8] analyzed the relationship between the stress transmission of blast waves and the thickness of the foamed concrete specimen. The results showed that when the thickness of foamed concrete specimen was greater than the critical thickness, the stress transmitted to the solid substrate would decrease; when the length of the specimen was less than this value, the stress transferred to the solid substrate increases. This critical length was related to the overpressure of the blast wave and the compressive strength of foamed concrete. Shang et al. [10] analyzed the fragmentation and energy absorption characteristics of foamed concrete cushioning layers with different thicknesses. The results showed that with the increase of the thickness of foamed concrete, the fractal dimension of fragmentation decreased, and the energy absorption efficiency increased first and then decreased. Tian et al. [11] discussed the influence of foamed concrete cushioning layers with different thicknesses on the deformation of aluminum plates. The results showed that the maximum deflection at the back of the aluminum plate decreased significantly with the increase of foamed concrete thickness, but the attenuation speed gradually slowed down. Wang et al. [12] discussed the protective effect of the thickness of foamed concrete on the steel box. The results showed that with the increase of the thickness of foamed concrete, the deformation of the steel box first decreased and then increased. When the thickness was less than a certain value, the deformation of the steel box was greater than that without foamed concrete. Li et al. [15] discussed the influence of the foamed concrete thickness on the acceleration response of the structures. The results showed that when the thickness was insufficient, the acceleration response of the structure becomes larger; if it was too thick, the blast resistance was not improved much. In application, the blast resistance and economic performance should be comprehensively considered to select the appropriate thickness. Zhou et al. [16] attributed the influence of foamed concrete thickness on the protective effects to the coupling results of three factors: the rapid compression of foamed concrete to reduce the stress, the energy absorption in the compression process, and the increase of incident energy caused by the reduction of explosion distance. The above research indicated that only when the coating thickness was greater than a critical value can the blasting damage be effectively reduced. However, the current protected objects are mostly metal equivalent models and tunnel structures. There are few studies on the blast resistance of the foamed concrete coated RC walls, and the influence mechanism of coating thickness on the blast resistance is not clear, resulting in the lack of theoretical guidance for relevant engineering applications.

In this paper, the cushioning effect of foamed concrete (density 820 kg/m^3^, water-cement ratio 0.4) on the blasting damage of a 120 mm RC wall was studied through blast experiments, numerical simulations, and shock wave theory. The influence of coating thickness on the stress distribution on the front side, the incident energy, and the spalling size on the back side of RC walls were discussed. The research results are expected to guide the engineering application of foamed concrete cushioning layers.

## 2. Microstructure and Mechanical Properties of Foamed Concrete

**Figure 1:**
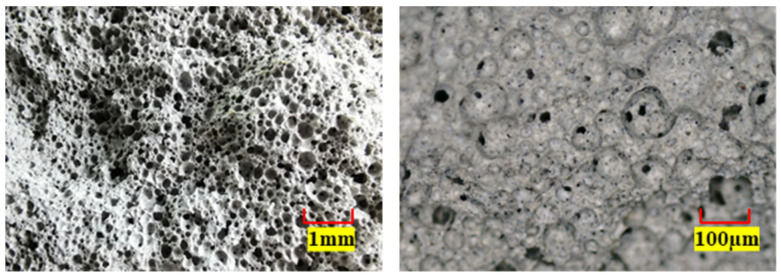
Microporous morphology of foamed concrete.

The foamed concrete (density 820 kg/m^3^) was prepared from cement, water, and foaming agent. The masses of the three materials were 800 kg, 320 kg, and 4 kg, respectively. The cement was ordinary Portland cement (P. O 42.5). The foaming agent was a kind of commercial polymer composite cement foaming agent. To produce foam, the foaming agent was diluted with water at a ratio of 1:40 by volume. Figure 1 shows the micropore morphology of foamed concrete specimens observed under a stereoscopic microscope. At low magnification, a large number of pores could be observed on the surface of the foamed concrete, and the specimen showed uneven surface characteristics. Increasing the magnification found that the pores were mostly closed cells. The diameters of those pores were different, and the wall thicknesses were much smaller than the diameters. The porous microstructure makes foamed concrete more fragile than ordinary concrete [6]. 

**Figure 2:**
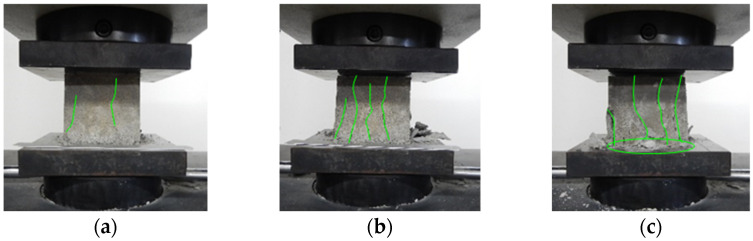
Quasi-static compression and failure process of foamed concrete: (**a**) Microcracks; (**b**) Vertical through cracks; (**c**) Large area cracks and bottom crushing.

**Figure 3:**
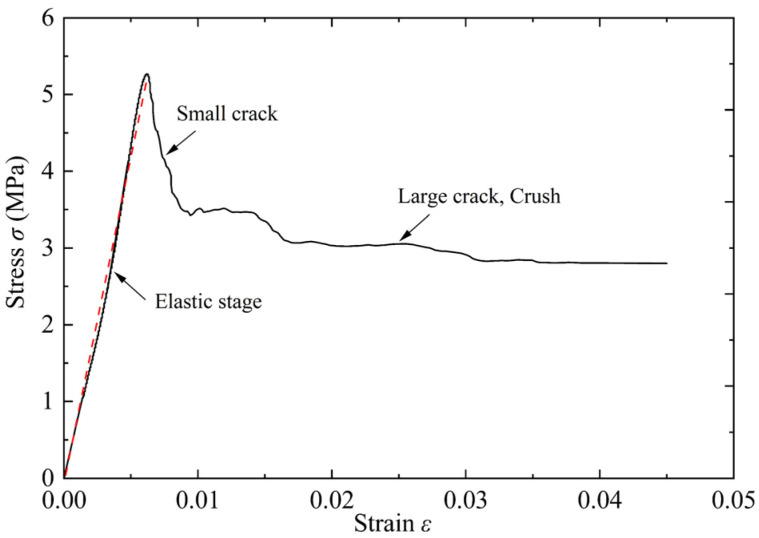
Quasi-static compression stress-strain curve of foamed concrete.

As shown in Figure 2, the quasi-static compression performance of foamed concrete was carried out on the universal testing machine. The size of the cube specimen was 100 mm × 100 mm × 100 mm, and the loading speed was 2 kN/s [17]. According to the results shown in Figure 2 and Figure 3, the quasi-static compression process of foamed concrete could be divided into three stages, named linear elastic response, microcrack propagation, and local crushing. With the propagation of microcracks, vertical cracks appear on the surface of the specimen, and the stress unloading process is linear at this stage. In the local crushing stage, the vertical crack has expanded into large through cracks, and the specimen is gradually compacted with the crushing of internal pores. At this stage, a long platform appears on the stress curve, indicating that foamed concrete has a long energy absorption stroke.

**Figure 4:**
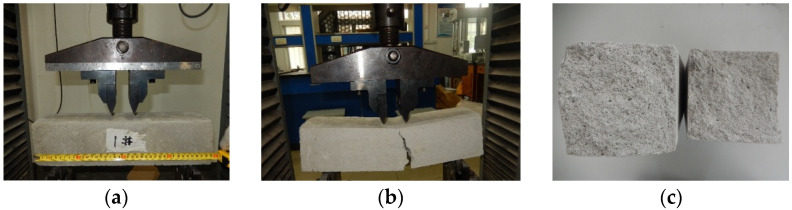
Four-point bending failure process of foamed concrete (**a**) Test layout; (**b**) Crack propagation; (**c**) Fracture morphology.

**Figure 5:**
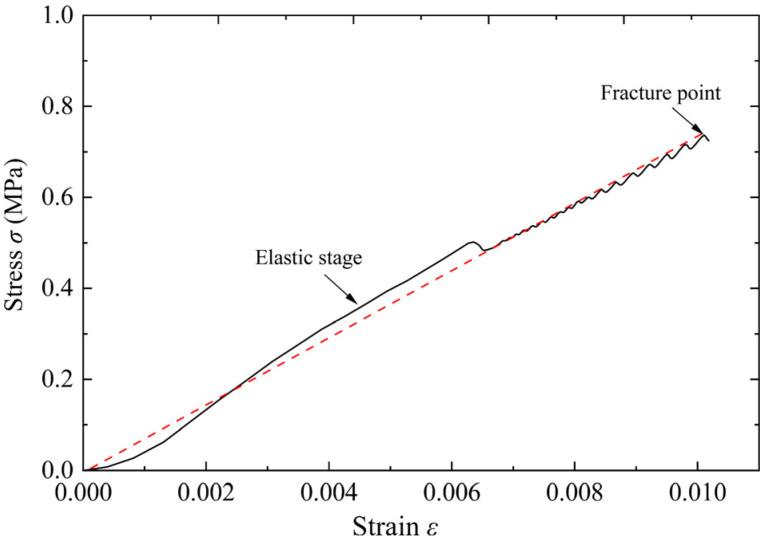
Four-point bending stress-strain curve of foamed concrete.

Similarly, the rectangular specimens with a size of 400 mm × 100 mm × 100 mm were prepared for the four-point bending test of foamed concrete. The loading speed of the testing machine was set to 0.2 kN/S [17]. Compared with the three-point bending test, the four-point bending test can characterize the bending resistance of materials through strength averaging, which is more suitable for heterogeneous materials. The fracture process of the specimen in the four-point bending test is shown in Figure 4. The crack propagates along the defect location, and the fracture surface is relatively flat. From the stress-strain curve shown in Figure 5, it can also be seen that the bending failure of foamed concrete has no obvious yield stage. When the bending limit is reached, the specimen suddenly breaks.

Some physical and mechanical parameters of foamed concrete are obtained according to the test results, as shown in Table 1. The porous structure of foamed concrete makes it fragile, so the foamed concrete is easy to fracture under blast load. The blasting energy is irreversibly absorbed in the fracture processes [10]. This is the basis of the blast resistance application of foamed concrete.

## 3. Methods

### 3.1. Blast Experiment

#### 3.1.1. Construction of Experimental Walls

**Figure 6:**
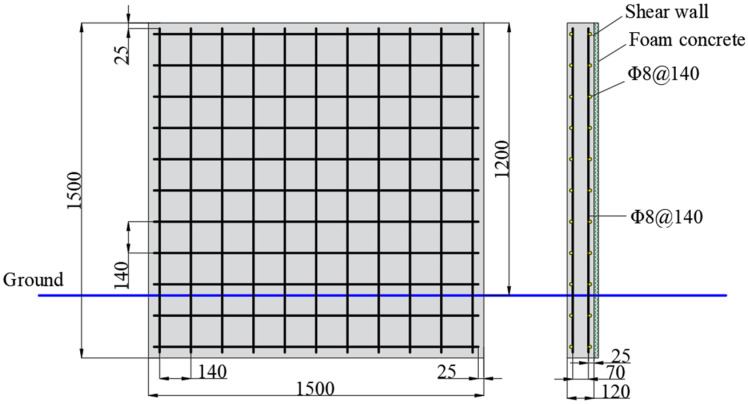
Structural dimension and reinforcement mode of the RC wall (unit: mm).

The experimental walls were constructed according to JGJ/T 341–2014 [18]. The structural dimension and reinforcement mode of the RC walls are shown in Figure 6. The RC wall had basic dimensions of 1500 mm × 1500 mm × 120 mm, of which the height of the aboveground part was set to 1200 mm, and the height of the underground part was set to 300 mm. The blue line shown in Figure 6 represented the ground. The concrete strength grade was C30. The reinforcement diameter was 8 mm, and the strength grade was HRB400. The reinforcement mode was double-layer and two-way reinforcement, and the reinforcement ratio was 0.614%. The spacing between two layers of reinforcement was 70 mm, and the spacing between layers of reinforcement was 140 mm. The thickness of the concrete cover was 25 mm. After constructing the RC walls, they were cured for 7 days under natural conditions. Then, the foamed concrete was uniformly coated on the surface of the walls. At this stage, the concrete strength reached 70% of the design strength [18]. To investigate the influence of foamed concrete thickness on the spalling resistance of RC walls, walls with different coating thicknesses were prepared. During the construction of concrete and foamed concrete, a certain number of specimens were reserved to verify the consistency of mechanical properties of materials. After the construction was completed, the walls continued to be cured under natural conditions for 28 days [18].

#### 3.1.2. Experimental Design and Layout

The blast experiments adopted a cylindrical TNT charge with a mass of 0.5 kg. The basic size of the charge was Φ100 mm × 41.5 mm. McVay [19] computed the critical wall thickness of shear wall spalling caused by naked charge as

(1) $$T=0.0730{\left(R/W^{1/3}\right)}^{-0.5856}W^{1/3},$$

where T is the wall thickness, R is the explosion distance, and W is the charge mass. Therefore, the critical explosion distance of spalling can be derived as

(2) $$R={\left(\frac{T}{0.0730\cdot W^{1/3}}\right)}^{-\frac{1}{0.5686}}\cdot W^{1/3}.$$

Substituting the mass of charge and the thickness of the shear wall, it can be calculated that R=221 mm.

**Figure 7:**
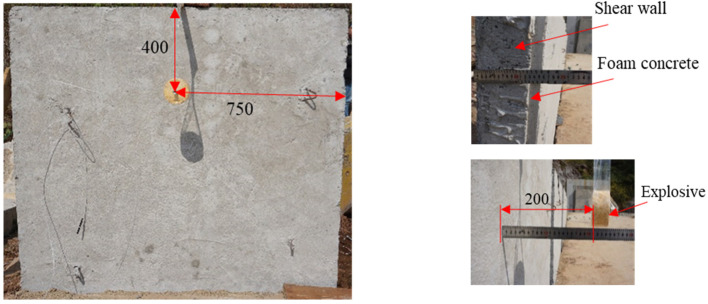
The layout of the blast experiments site (unit: mm).

In the blast experiments, the charge was located at the center of the wall in the horizontal direction, and 400 mm away from the top of the wall in the vertical direction. The stand-off was set to 200 mm. According to the calculation results of Equation (2), the RC wall is in a critical spalling state under this working condition. Three blast experiments were carried out for three types of RC walls, namely, the wall uncoated (SW), the wall coated with 10 mm foamed concrete (10 mm FC + SW), and the wall coated with 20 mm foamed concrete (20 mm FC + SW). SW was taken as the reference that corresponds to the spall damage mode of the wall. Figure 7 shows the site layout of the blast experiments. The side near the explosive is the front side of the RC wall and away from the explosive is the back side of the RC wall.

### 3.2. Numerical Simulation

#### 3.2.1. Finite Element Model

**Figure 8:**
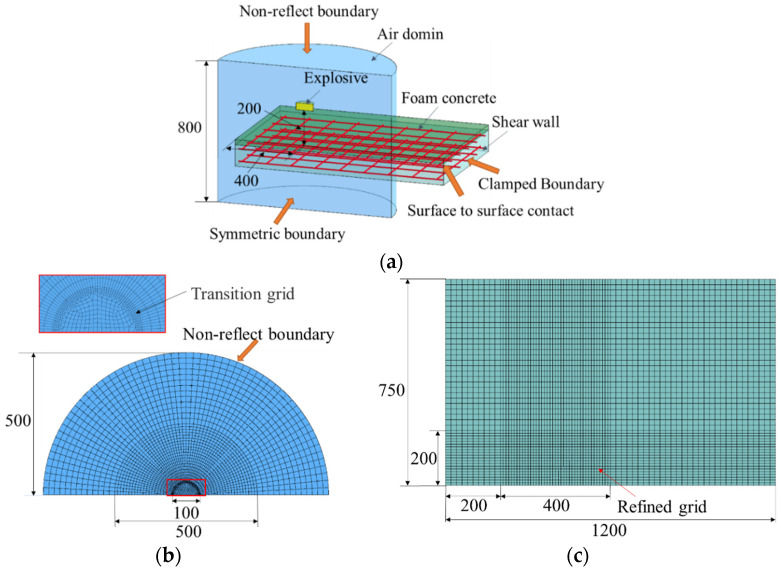
Model and grid meshing (unit: mm): (**a**) Finite element model; (**b**) Air domain grid; (**c**) RC wall grid.

The explicit nonlinear finite element program LS-DYNA was used to simulate the deformation and damage of the RC walls under blast loading [12,20]. As shown in Figure 8a, the finite element model comprised four parts, namely, the air domain, explosive, RC wall (steel bar and concrete), and foamed concrete. Given the face symmetry of the structure, to reduce the amount of calculation, the model was simplified by establishing a one-half model with symmetrical constraints [20]. The underground part was omitted from this model. Therefore, the RC wall model had an overall size of 750 mm × 1200 mm × 120 mm. The beam element was selected for the steel bar, and the solid element was selected for the other parts [20]. The air and explosive adopted the Euler grid, whereas the concrete and foamed concrete adopted the Lagrange grid. To achieve the best match among grid quality, structural size, and solution accuracy, the central densified grid division method was adopted for the air domain and concrete with the charging axis as the center. The grid length of the air domain measured 5, 10, and 20 mm from the center to the outside, whereas the concrete grid length measured 10 mm and 20 mm from the center to the outside. The size of the grid densified area is shown in Figure 8b,c. In terms of contact, the Arbitrary–Lagrange–Euler (ALE) approach was utilized to model the interface between the air and structure. Given the very short duration of blast load, the relative slip between reinforcement and concrete was not considered, and they were connected by a common node [21]. The surface-to-surface contact was applied to the interface between the concrete and foamed concrete. In terms of constraints, a non-reflect boundary was defined around the air domain to eliminate the influence of the reflected rarefaction wave on the calculation results [12]. The fixed support constraint was adopted at the bottom of the RC wall to reflect the constraint effect of the foundation on the wall.

#### 3.2.2. Material Models and Parameters

Air can be regarded as an ideal gas, which was described using the NULL (MAT_009) constitutive model and the LINEAR_POLYNOMIAL (EOS_001) equation of state [22]. The pressure p can be expressed as:

(3) $$p=\left(\gamma -1\right)\frac{\rho }{\rho_{air}{}_{0}}E,$$

where γ is the adiabatic index, $\rho_{air}{}_{0}$ is the initial density of air, and E is the specific internal energy. The main parameters of air are shown in Table 2.

The initial parameters and detonation pressure of TNT explosive were described using the HIGH_EXPLOSIVE_BURN (MAT_008) constitutive equation and the JWL (EOS_002) state equation [22]. The calculation of detonation pressure can be expressed as follows:

(4) $$p=A\left(1-\frac{\omega \eta }{R_{1}}\right)e^{-\frac{R_{1}}{\eta }}+B\left(1-\frac{\omega \eta }{R_{2}}\right)e^{-\frac{R_{2}}{\eta }}+\omega \eta E,$$

where $\eta =\rho /\rho_{0}$, $\rho_{0}$ is the initial density of explosive, and ρ is the density of detonation product. The pressure of the blast wave decreases with the decrease of η during the expansion of detonation products. Values of constants A, B, $R_{1}$, $R_{2}$, and ω were determined from dynamic experiments. The specific values of the above parameters can be seen in Table 3.

An RHT (MAT_272) model was used to analyze concrete structures subjected to impulsive loadings [23]. Considering the compressibility of concrete under high-pressure impact, the RHT model defines the porosity variable to describe the Hugoniot curve and compaction relationship of concrete. Porosity decreases with the increase of impact pressure. When spalling occurs in concrete structures, the tensile strength, fracture energy, and strain rate in tension are very important [24]. The RHT model describes the variation law of initial yield strength, failure strength, and remaining strength of concrete by introducing three limit surfaces named elastic limit surface, failure surface, and remaining strength surface. The strength model comprehensively considers the mechanical characteristics of concrete, such as the stress hardening, strain hardening, strain rate hardening, dependence of tension-compression Meridian on the third invariant, and cumulative damage (strain softening). The failure surface equation of the RHT model is:

(5) $$\sigma_{eq}^{*}\left(p,\theta ,\dot{\varepsilon }\right)=Y_{TXC}^{*}\left(p\right)R_{3}\left(\theta \right)F_{rate}\left(\dot{\varepsilon }\right)$$

where $Y_{TXC}^{*}\left(p\right)$ is the compression meridian and $F_{rate}\left(\dot{\varepsilon }\right)$ is the strain rate enhancement factor. $R_{3}\left(\theta \right)$ is the corner function, which is used to describe the relationship between concrete failure strength and stress tensor invariants [24]. The damage parameter is accumulated with plastic strain according to:

(6) $$D=\int_{\varepsilon_{p}^{h}}^{\varepsilon_{p}}\frac{d\varepsilon_{p}}{\varepsilon_{p}^{f}}$$

For LS-DYNA post-processing, set NEIPH = 4 in DATABASE_EXTENT_BINARY to observe the damage to concrete materials [25]. The main parameters of concrete can be found in Table 4.

A PLASTIC_KINEMATIC (MAT_003) model was adopted to describe the reinforcement. This model considers the strain rate effect of materials and is suitable for calculating the deformation of beam elements under high pressure and high strain rate [26]. The main parameters of reinforcement are shown in Table 5

The CRUSHABLE_FOAM (MAT_063) model was characterized the compressibility of foamed concrete through volumetric strain [25]. The strain rate effect of foamed concrete was not considered in this model. The compression stress-strain curve, the density, and Young’s modulus were from the test results in Section 2. Other parameters were from references [12,27]. The specific parameters are shown in Table 6.

#### 3.2.3. Mesh Convergence Verification

**Figure 9:**
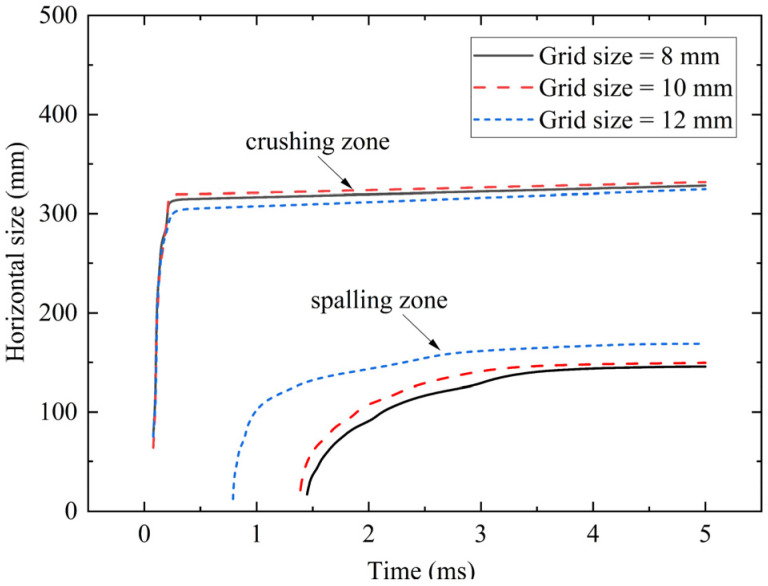
Mesh convergence verification.

As the damage mainly occurs in the mesh refinement area, the grid size of the refinement area was set as 8 mm, 10 mm, and 12 mm respectively, and the horizontal damage sizes of the foamed concrete and the RC wall for the 20 mm FC + SW wall experiment are calculated. The results are shown in Figure 9. With the increase of time, the calculation results corresponding to the three meshes tend to be horizontal. At 5 ms, the crushing sizes of the foamed concrete are 322 mm, 326 mm, and 320 mm respectively. When the grid size is 10 mm, the errors with the other two sizes are 1.24% and 1.84%. The spalling sizes of the RC wall are 142 mm, 146 mm, and 162 mm respectively. When the grid size is 10 mm, the errors with the other two sizes are 2.74% and 10.96%. The experimental results of the two parameters are 330 mm and 138 mm, and the errors of the 10 mm grid are 1.82% and 5.80%. Based on the above analysis, when the grid size of the mesh refinement area is 10 mm, the damage calculation results meet the convergence requirements.

## 4. Experimental and Simulation Results

**Figure 10:**
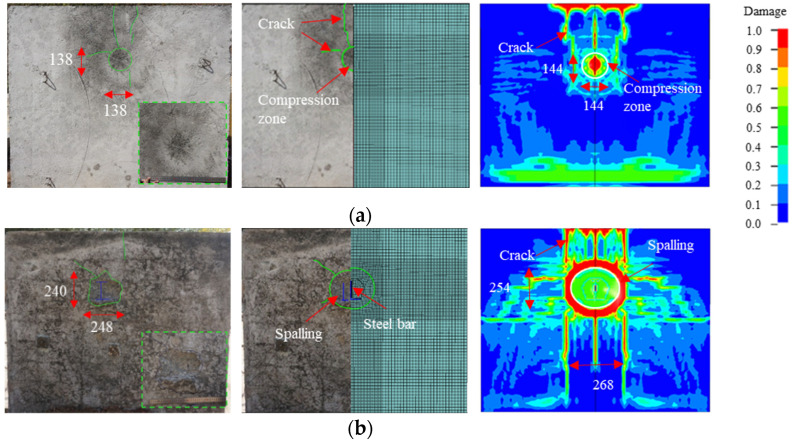
Experimental and simulation results of the damage of the SW wall (unit: mm): (**a**) Front side; (**b**) Back side.

Figure 10 shows the experimental and simulation results for the overall damage to the SW wall. One can see from the figure that the SW wall had a spall damage mode. An obvious compression zone with a diameter of 138 mm can be seen on the front face of the wall. Several radial cracks with lengths of 200 mm to 400 mm were distributed around the compression zone, and some of these cracks extended from the top to the back of the wall. A spalling pit was also found on the back side of the wall. Under the influence of reinforcement distribution and boundary conditions, the spalling pit was approximately elliptical with a size of 248 mm × 240 mm, and the maximum spalling depth was 40 mm. Some of the reinforcement was exposed in the pit, the maximum exposed length was 168 mm, and no obvious bending of reinforcement was observed. Radial cracks were also distributed around the spalling pit, and the cracks on the top were connected to the crack on the front face of the wall. The simulation results also revealed spalling and exposed reinforcement and a similar distribution of cracks. Crack propagation and bifurcation can also be observed, and a tensile damage zone appeared at the top of the wall. The size of the simulated spalling pit was 268 mm × 254 mm, and the spalling depth was 42 mm. The relative errors with the experimental results were 14.4% and 5%, respectively.

**Figure 11:**
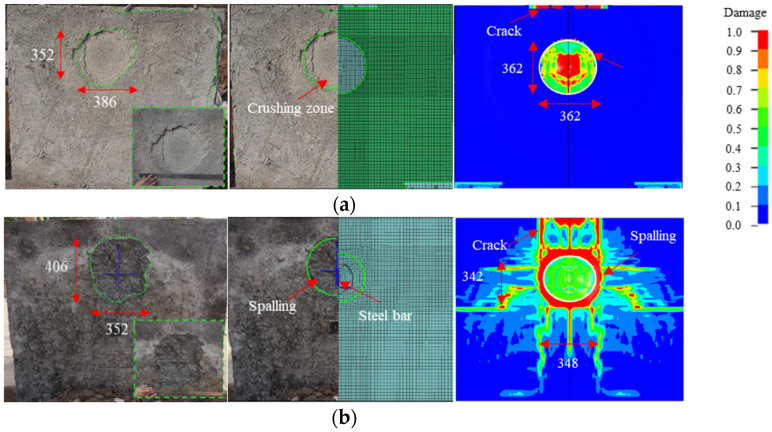
Experimental and simulation results for the damage of the 10 mm FC + SW wall (unit: mm): (**a**) Front side; (**b**) Back side.

Figure 11 shows the experimental and simulation results for the overall damage to the 10 mm FC + SW wall. This wall also had a spall damage mode, but the spalling area on the back side of the wall was enlarged. A crushing zone with a planar size of 440 mm × 352 mm and a depth of 10 mm was observed in the foamed concrete layer. Many cracks were observed in the foamed concrete layer, and some foamed concrete fragments flew off the edge of the wall. A partial separation was also observed between the foamed concrete layer and the RC wall, and no damage was found on the front side of the RC wall. The size of the spalling area on the back side of the RC wall was 352 mm × 406 mm, whereas the maximum spalling depth was 45 mm. Several radial cracks with a length ranging from 50 mm to 200 mm surrounded the spalling area. Three exposed reinforcements were identified, and the maximum exposed length was 286 mm. Results of the numerical simulation also revealed an enlarged spall on the back side of the 10 mm FC + SW wall. The crushing zone had a planar size of 362 mm × 362 mm, leading to a 3.6% error with the experimental results. The size of the spalling area was 348 mm × 342 mm and the maximum spalling depth was 48 mm, which indicated relative errors of 16.7% and 6.7% with the test results, respectively.

**Figure 12:**
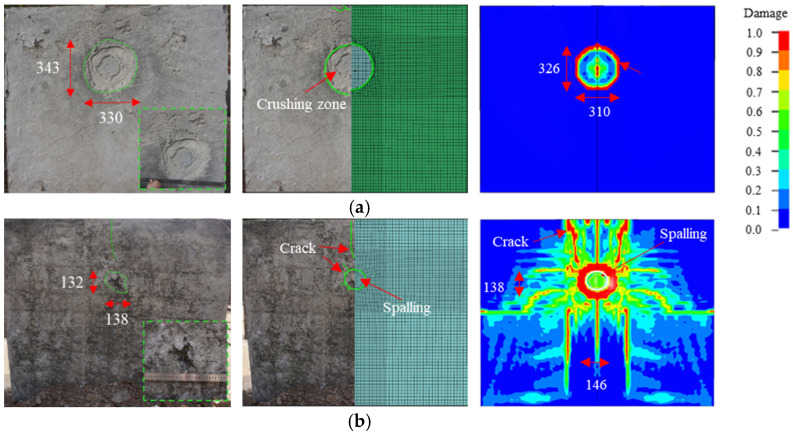
Experimental and simulation results for the damage of the 20 mm FC + SW wall (unit: mm): (**a**) Front side; (**b**) Back side.

Figure 12 shows the experimental and simulation results for the overall damage to the 20 mm FC + SW wall. The spalling area on the back side of the wall was significantly reduced. The crushing mode of foamed concrete was layer by layer, and the inner and outer crushing zones had an obvious boundary. The crushing zone had a planar size of 330 mm × 343 mm and a depth of 20 mm. A small amount of foamed concrete fragments flew out at the boundary. The foamed concrete layer and RC wall were partially separated, and the compression zone of the RC wall was enlarged. However, the damage was observed only on the surface. A small area of spalling was observed on the back side, and several radial cracks with a length ranging from 50 mm to 200 mm surrounded the spalling area. The spalling area had a size of 138 mm × 132 mm and a maximum spalling depth of 20 mm. No exposed reinforcement was found. In the numerical simulation, the planar size of the crushing zone was 326 mm × 310 mm, representing a 10.7% error from the experimental results. Meanwhile, the size of the spalling area was 146 mm × 138 mm and the maximum spalling depth was 22 mm, representing relative errors of 10.6% and 10% with the experimental results, respectively.

When the blast load interacts with the RC wall, the wall is subjected to a compression wave on the loading surface while subjected to a tensile wave on the free surface [28]. Given that its tensile strength is far less than its compressive strength, concrete experiences spalling when the strength of the tensile wave exceeds the dynamic tensile strength [29]. The RC wall studied in this paper was a cantilever structure. The compression pit could be observed on the front of the walls, and the spalling phenomenon could be observed on the back, top, and side of the walls. Table 7 shows the main damage parameters of the three kinds of walls obtained from the experiments and simulations. According to the data in Table 7, the damage sizes of RC walls are much smaller than their plane sizes (1200 mm × 1200 mm). Therefore, the influence of boundary conditions on damage sizes can be ignored, and the results in this paper can also apply to other boundary conditions. Radial cracks are distributed near the damage zone, and branching occurs during crack propagation [30,31]. The damage phenomenon of RC walls under different foamed concrete coating thicknesses was well described by experiments and simulations. Both the experimental and simulation results showed that when the coating thickness of foamed concrete was insufficient (10 mm), the damage to the RC wall expands; when the coating thickness of foamed concrete was thicker (20 mm), the damage to the wall decreased. These phenomena were similar to those observed by Tian [11] and Zhou [16] in the metal equivalent target experiments.

## 5. Discussion 

### 5.1. Blast Resistance of Foamed Concrete-Coated RC Walls

**Figure 13:**
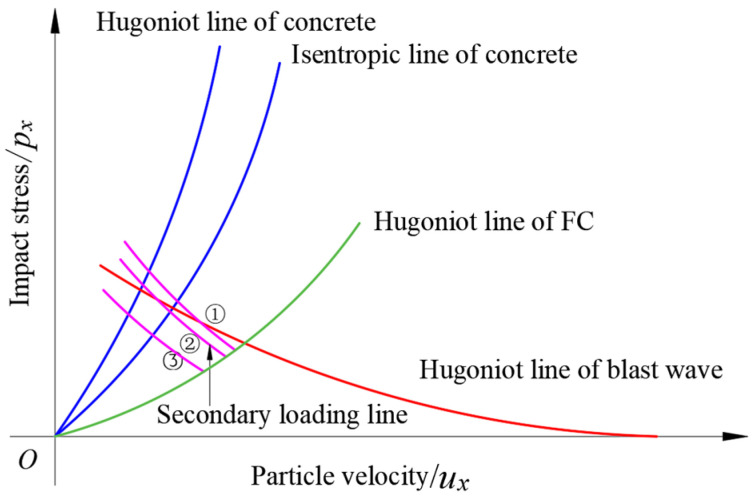
The loading process of the blast wave on the foamed concrete-coated RC walls.

The initial state parameters of the impact point among the blast wave, foamed concrete, and RC wall can be calculated through the mirror inversion method [32]. The solving process is based on the following hypotheses [32]: (1) the Hugoniot line of the left propagating wave is symmetrical to that of the right propagating wave; (2) the Hugoniot line of the secondary loading can be replaced by that of the first loading; (3) the state points of blast wave attenuation in foamed concrete is distributed on the Hugoniot line. As shown in Figure 13, the interaction process between blast wave and foamed concrete- coated RC wall can be divided into three stages: (1) blast wave is loaded on the foamed concrete; (2) Blast wave propagates and attenuates in the foamed concrete layer; (3) Blast wave passes through the foamed concrete layer and is loaded on the RC wall.

The intersection points among the state lines of the blast wave, foamed concrete, and concrete represent the initial state parameters of the interface. When the materials are determined, the initial state parameters are only related to the intensity of blast load. Under the same blast load, the initial stress of foamed concrete is lower than that of concrete. When the blast load increases (such as a smaller stand-off or a larger charge mass), the initial stress will increase. The propagation and attenuation of blast waves in foamed concrete are very complex. On the one hand, under the high-speed impact, foamed concrete will be compacted, and the transmitted stress will rise rapidly; on the other hand, the fragmentation process of foamed concrete will absorb energy and reduce stress [8]. The thickness of foam concrete mainly affects the attenuation of blast wave and the fragmentation of foamed concrete [8,10]. According to hypothesis (3), after the blast wave passes through the foamed concrete layer, the state point is still on its Hugoniot line. Combined with hypotheses (1) and (2), the secondary loading process of the blast wave from foamed concrete to the RC wall can be obtained. It can be seen from Figure 13 that, when the coating thickness is thin, the stress attenuation is very small, and the state points of the secondary loading of isentropic and adiabatic compressions are both higher than those of the blast wave direct loading (curve ①). The impact stress on the concrete of secondary loading is greater than that of blast wave direct loading. The attenuation increases with the increase of coating thickness. One possibility is that the state point is adiabatic compression for the blast wave to directly load on the concrete, whereas isentropic compression through secondary loading (curve ②). At this time, the impact stress of secondary loading is lower than that of the direct loading, while the particle velocity of secondary loading is still greater than that of the direct loading. Only when the coating thickness reaches certain values, do the state points of the secondary loading of isentropic and adiabatic compressions lower than those of the blast wave direct loading (curve ③). Both the impact stress and the particle velocity of secondary loading are smaller than that of direct loading. Theoretical analysis shows that there is also a critical coating thickness in foamed concrete coated RC structures. The critical coating thickness is related to the load intensity of blast wave, the high-pressure state equation of foamed concrete, and the energy absorption ability of foamed concrete.

### 5.2. Attenuation of the Blast Wave in Foamed Concrete

In the simulation results, taking the loading center of the explosion wave as the origin (x=0), the stress attenuation of the foamed concrete layer in the thickness direction was monitored. The attenuation law can be fitted by Equation (7) [33]:

(7) $$\sigma =\sigma_{0}\cdot e^{-\alpha t}$$

where $\sigma_{0}$ is the initial stress on the surface of the foamed concrete layer, α is the attenuation coefficient of the blast wave in foamed concrete, and t is the thickness of the foamed concrete. 

**Figure 14:**
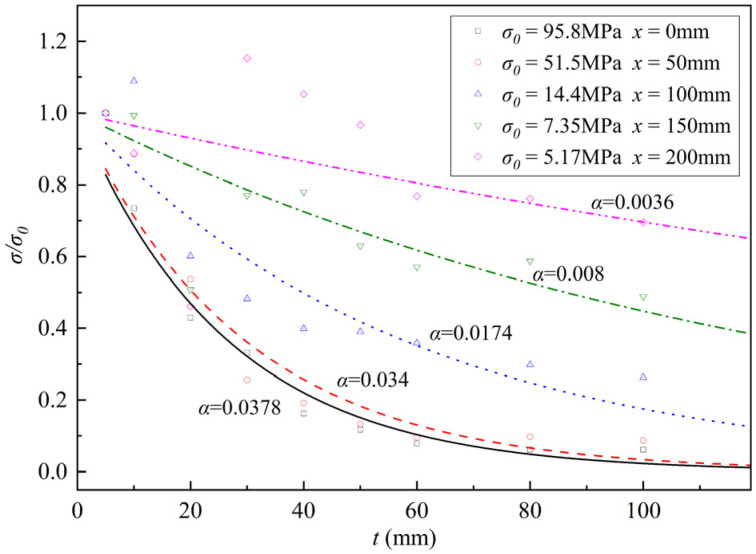
Stress attenuation of foamed concrete at different loading positions.

The stresses under different coating thicknesses were dimensionless, and the attenuation law of normalized stress at different locations was fitted using Equation (7). As shown in Figure 14, the attenuation of blast waves in foamed concrete follows the exponential attenuation law, and the attenuation exponents decrease as the blast wave moves away from the loading center, thereby indicating that the attenuation rate of the blast wave is related to the initial stress. This is because when the initial stress is high, the fragmentation degree of foamed concrete is higher, and more energy is absorbed due to fragmentation [10]. In addition to increasing the coating thickness of foamed concrete, doping fiber [34,35,36], using composite structures [37,38,39] or higher density [11], can also improve the energy absorption ability of foamed concrete.

### 5.3. Incident Stress and Energy on the Front Side of RC Walls

**Figure 15:**
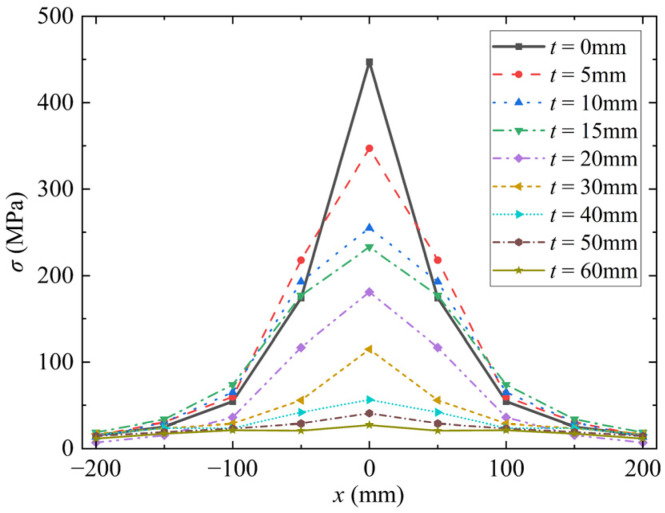
Stress distribution on the front side of RC walls with different coating thicknesses.

Nian et al. [8] and Li et al. [15] found that the dynamic responses of foamed concrete composite structures are related to the coating thickness. The blast response of RC walls with different coating thicknesses of foamed concrete was simulated under the experimental loading conditions. The statistical results are shown in Figure 15. The peak stress in the loading center is the largest and decreases gradually along the radial direction. After coated with foamed concrete, the peak stress near the loading center decreases with the increase of the coating thickness of foamed concrete, whereas the peak stress near the outside first increases and then decreases with the increase of the thickness of foamed concrete. The above results also prove that the stress attenuation is the fastest in the loading center (due to high pressure). As shown in Figure 15, when the coating thickness of foamed concrete is 5, 10, and 15 mm, the peak stress in some areas is greater than that of the uncoated wall; when the coating thickness is greater than 15 mm, the peak stress is smaller than that of the uncoated wall at all positions. The trend of transmitted stress with coating thickness reflected in Figure 15 is consistent with the conclusions from Nian et al. [8] and Li et al. [15]. Therefore, from the calculation results of transmitted stress, it can be obtained that the critical coating thickness is 15 mm.

**Figure 16:**
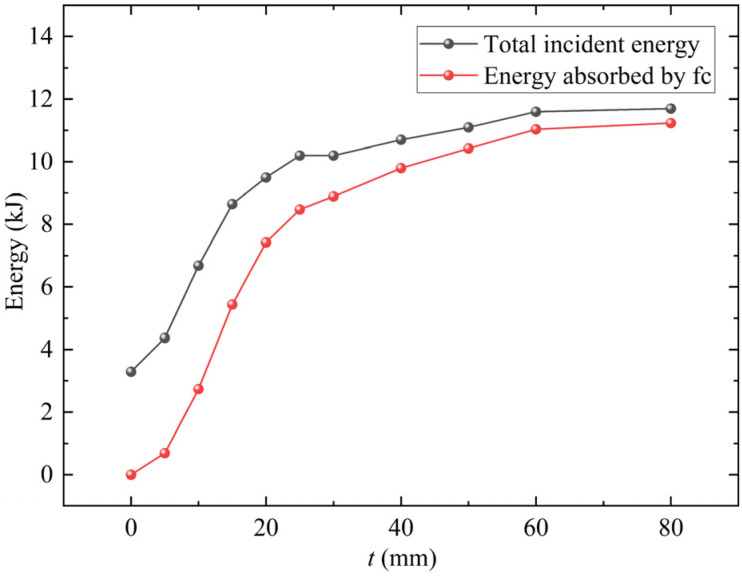
The variation of total incident energy and energy absorbed by foamed concrete with coating thickness.

Wang et al. [12] analyzed the protection effectiveness of foamed concrete on the steel plate from the perspective of energy and found that the influences of foamed concrete thickness on the incident energy and residual deformation of the steel plate are consistent. In the foamed concrete coated RC walls, the blasting energy is absorbed by the foamed concrete layer and the RC wall successively. According to the stress wave theory, since the impedance of foamed concrete is lower than that of ordinary concrete, the total incident energy of RC wall coated with foamed concrete increases with the impact compression process of Foamed concrete [32]. Figure 16 reflects the variation of total incident energy and energy absorbed by foamed concrete with coating thickness. When the coating thickness of foamed concrete is thin, the energy absorbed by the fragmentation of foamed concrete is insufficient to offset the increment of total incident energy [12].

**Figure 17:**
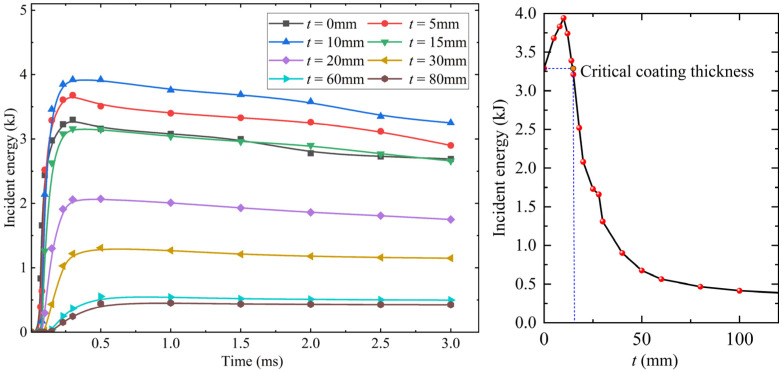
Energy incident into RC walls with different foamed concrete coating thicknesses.

Figure 17 shows the energy incident to the RC wall under different foamed concrete coating thicknesses. With the increase of the coating thickness, the rise time of the energy incident into the RC wall becomes longer, and the peak first increases and then decreases. When the coating thickness is 15 mm, the incident energy of the RC wall is close to that without foamed concrete coating. Therefore, it can also predict the critical coating thickness from the calculation results of incident energy. 

### 5.4. Damage of Foamed Concrete-Coated RC Walls

**Figure 18:**
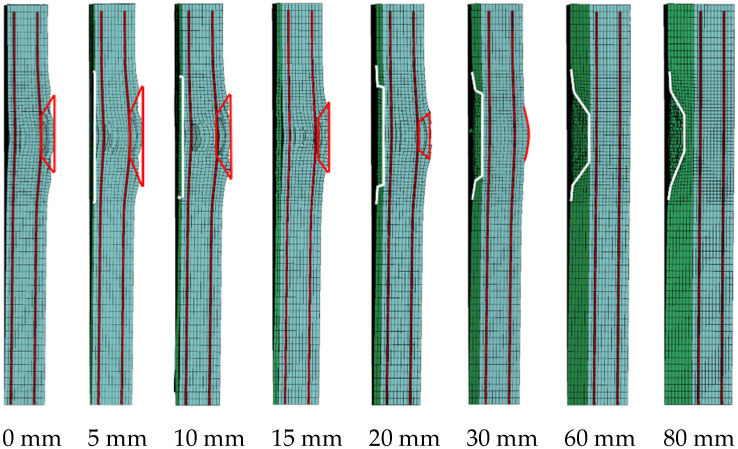
Typical damage results for the RC walls with different coating thicknesses.

Tian et al. [11] and Wang et al. [12] found that choosing an appropriate coating thickness of foamed concrete could reduce the deformation of the metal equivalent target. Figure 18 shows the typical damage of RC walls with different foamed concrete coating thicknesses. With the increase of the foamed concrete coating thickness, the spalling damage on the back side of the RC wall first increases and then decreases compared with the wall without coating (red line), and two turning points named spalling reduction and no spalling appear in sequence. The failure mode of foamed concrete layer also shows a transitional change from direct shear failure to compression shear failure (white line) with the increase of coating thickness. 

**Figure 19:**
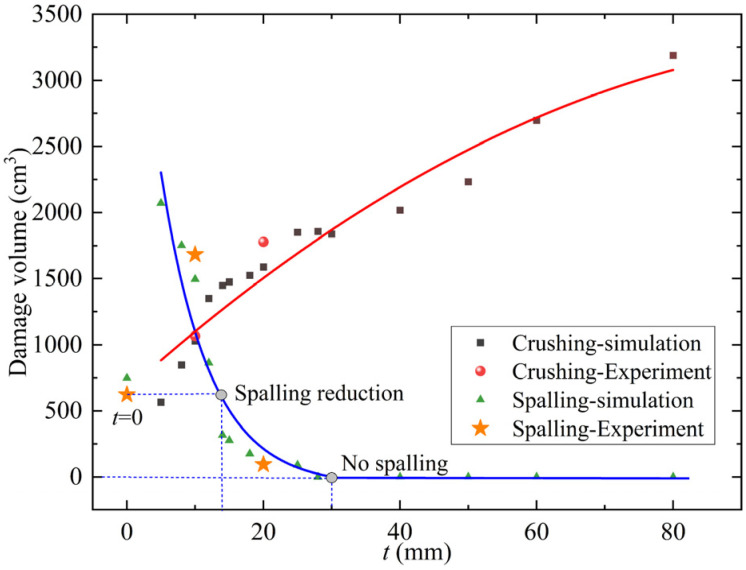
Experimental and simulation results of the volume of crushing and spalling zones.

The volume of foamed concrete crushing and RC wall spalling zones was calculated according to the experimental and simulation results. The calculation results are shown in Figure 19. With the increase of foamed concrete coating thickness, the volume of the crushing zone increases, but the growth rate slows down and gradually tends to a limited value (red line). The calculation results of the crushing zone show that the energy absorbed by foamed concrete under a certain load is limited. When the coating thickness exceeds a certain value, it is difficult to greatly improve the energy absorption of foamed concrete. The spall damage of the RC wall first increases and then decreases with the increase of coating thickness (blue line). When the coating thickness is less than 15 mm, the spalling volume is larger than the RC wall without coating; when the coating thickness is 15–30 mm, the spalling volume is smaller than that without coating; When the coating thickness is greater than 30 mm, no spalling appears on the back side of the RC wall. Therefore, for a 120 mm RC wall in the critical blasting spall, the cushioning thickness of foamed concrete corresponding to spalling reduction is 15 mm. The damage calculation results are consistent with the critical coating thickness of foamed concrete predicted by incident stress and energy. In other words, when the intensity of the blast load, the energy absorption capacity of foamed concrete, and the bearing capacity of the structure are determined, the critical coating thickness of foamed concrete can be predicted.

The critical coating thickness in this paper is obtained under the critical blasting spall of a 120 mm RC wall, which is the minimum thickness of the foamed concrete cushion. When a smaller stand-off or a larger charge mass is used, the critical coating thickness will increase correspondingly due to the increase of incident stress and energy [22]. In addition, when the energy absorption capacity of the foamed concrete is improved, the critical coating thickness will decrease correspondingly. When the coating thickness is greater than the lower critical thickness, the damage to the RC wall continues to decrease. However, the coating thickness cannot be increased without a limit, for the energy absorption efficiency will be reduced if it is too thick [10]. It should be comprehensively considered the blast resistance, economy, and space to select the appropriate coating thickness in the blast resistance design [15].

## 6. Conclusions

This paper studied the influence of the coating thickness of foamed concrete (density 820 kg/m^3^, water-cement ratio 0.4) on the blast resistance of a 120 mm RC wall. The results of this work can provide a reference for the blast resistance designs of any type of RC walls and panels. The main conclusions are as follows:

(1) The influences of foamed concrete coating on the blast resistance of RC walls include: Due to the impedance of foamed concrete is low, the initial stress of the structure is reduced; after high-speed compression, foamed concrete enters the compacted state, and the stress on the surface of RC wall increases significantly when the coating thickness is insufficient; and the fragmentation process of foamed concrete absorbs the energy of stress wave, and the transmitted stress gradually attenuates.
(2) Coating thickness is one of the important factors that affect the attenuation of the blast wave in foamed concrete. The attenuation of stress is exponential with the increase of coating thickness. The attenuation index increases with the increase of load intensity.
(3) Foamed concrete coated RC wall has a lower critical coating thickness. When the thickness of foamed concrete is less than that value, the blast resistance of the RC wall decreases; when it is greater than that value, the blast resistance of the RC wall is gradually improved as the coating thickness increases. The lower critical coating thickness can be predicted by monitoring the explosive stress and energy incident to the RC wall.
(4) For the given 120 mm RC wall, the lower critical thickness of the foamed concrete coating to enhance the blast resistance is 15 mm. In the blast resistance design, the coating thickness of foamed concrete should not be less than the lower critical thickness, and the optimum coating thickness can be determined by comprehensively considering the blast resistance, economy, and space.

## Figures and Tables

**Table 1:** Main physical and mechanical parameters of foamed concrete.

Density (kg/m^3^)	Yang’s Modules (GPa)	Compressive Strength (MPa)	Platform Stress (MPa)	Flexural Strength (MPa)
820	880	5.28	2.82	0.74

**Table 2:** Material parameters of air.

Density (g/cm^3^)	Sound Velocity (m/s)	Temperature (K)	Adiabatic Index	Initial Specific Internal Energy (J/kg)
1.225 × 10^−3^	344	288	1.4	2.068 × 10^5^

**Table 3:** Material parameters of TNT explosive.

Density (g/cm^3^)	Detonation Velocity (m/s)	C-J Pressure (GPa)	*A* (Gpa)	*B* (Gpa)	*R*_1_	*R*_2_	*ω*
1.53	6641	18.5	329.92	3.3	4.15	0.9	0.35

**Table 4:** Material parameters of concrete.

Density (g/cm^3^)	Compressive Strength (MPa)	Tensile Strength (MPa)	Shear Modulus (GPa)	*A*	*N*
2.38	32.7	5.85	16.7	1.6	0.61

**Table 5:** Material parameters of reinforcement.

Density (g/cm^3^)	Young’s Modulus (MPa)	Poisson Ratio	Yield Stress (MPa)	Tangent Modulus (MPa)
7.80	210	0.3	414	1300

**Table 6:** Material parameters of foamed concrete.

Density (g/cm^3^)	Young’s Modulus (MPa)	Poisson Ratio	Tensile Stress Cutoff (MPa)	Damping Coefficient
0.82	880	0.1	0.64	0.1

**Table 7:** Damage parameters of foamed concrete-coated RC walls.

Coating Thickness (mm)	Crushing Size (mm)		Spalling Size (mm)		The Exposed Length of Reinforcement (mm)
	Experiment	Simulation	Experiment	Simulation	
0	-	-	248 × 240 × 40	268 × 252 × 42	168
10	386 × 352 × 10	362 × 362 × 10	352 × 406 × 45	348 × 342 × 48	286
20	330 × 343 × 20	326 × 310 × 20	138 × 132 × 20	146 × 138 × 22	-

## Data Availability

Data are contained within the article.
